# Mitral Valve Endocarditis due to *Lactobacillus*

**DOI:** 10.1155/2018/8613948

**Published:** 2018-09-04

**Authors:** Patrick Groga-Bada, Iris I. Mueller, Federico Foschi, Meinrad Gawaz, Christian Eick

**Affiliations:** ^1^University Hospital, Department of Cardiology and Cardiovascular Medicine, Eberhard Karls University Tuebingen, 72076 Tuebingen, Germany; ^2^University Hospital, Department of Gastroenterology and Hepatology, Eberhard Karls University Tuebingen, 72076 Tuebingen, Germany

## Abstract

*Lactobacillus* species are Gram-positive, facultative anaerobic, rod-shaped bacteria. They belong to the lactic acid bacteria group and are also known as a usual part of the normal flora of the gastrointestinal tract as well as of the urinary and genital tracts. They are an infrequent human pathogen but can induce several infections such as bacteremia and infectious endocarditis. We report the case of an 81-year-old woman with *Lactobacillus bacteremia* and mitral valve endocarditis as well as splenic abscesses.

## 1. Introduction

*Lactobacillus* species are Gram-positive, facultative anaerobic, rod-shaped bacteria [1, 2]. They belong to the lactic acid bacteria group and are also known as a usual part of the normal flora of the gastrointestinal tract as well as of the urinary and genital tracts [2, 3]. The occurrence of those bacteria in miscellaneous mucosa is normally associated with stimulation of the immune system and protection against pathogens [4]. They are an infrequent human pathogen but can induce several infections [5] such as bacteremia, infectious endocarditis, dental caries, and intra-abdominal infections with liver or splenic abscesses [6]. Endocarditis due to *Lactobacillus* is associated with structural heart disease, recent surgery, extended antibiotic and probiotic use, reduced immunity, and other relevant comorbid factors [7, 8]. We report a case of *Lactobacillus bacteremia* with mitral valve endocarditis and splenic abscesses.

## 2. Case Presentation

**Figure 1:**
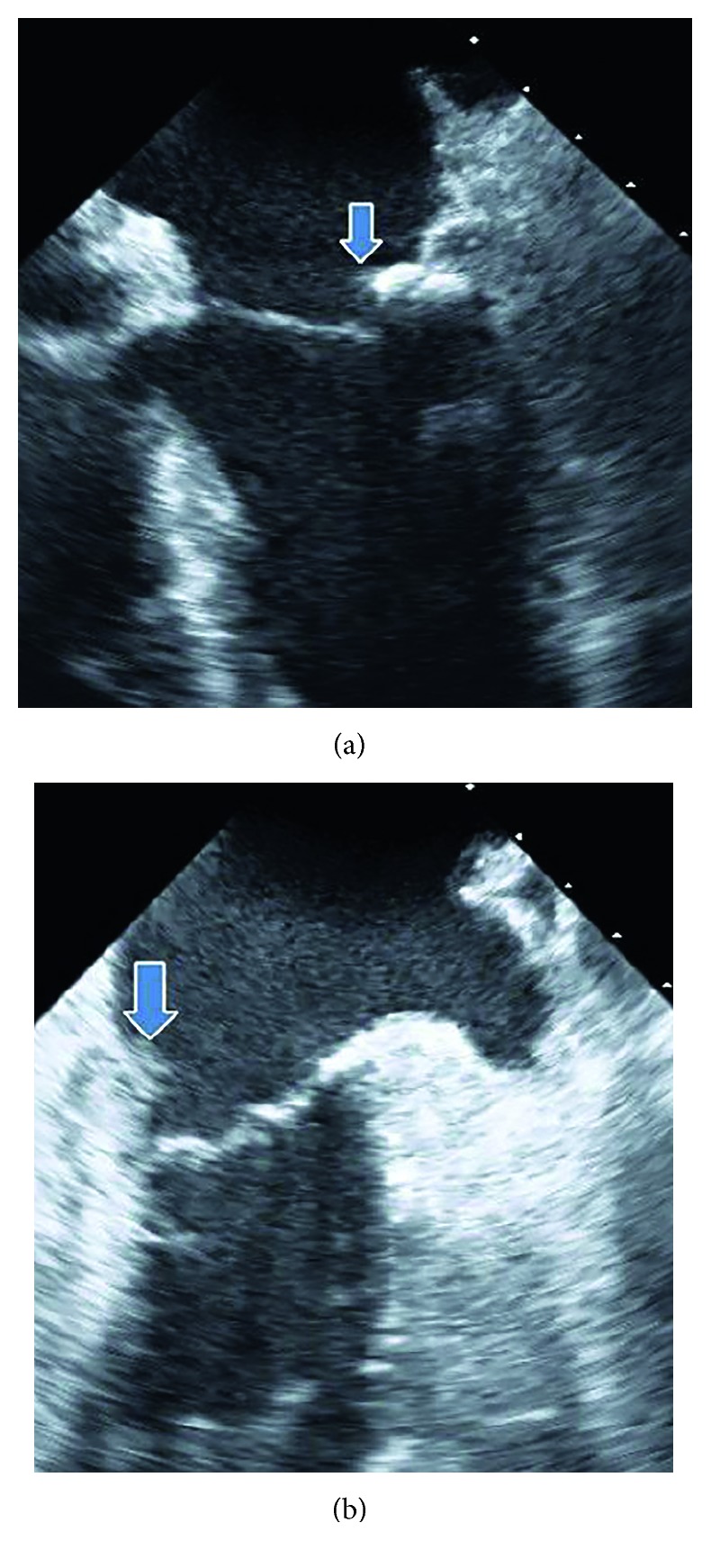
(a) Transesophageal echocardiography with a corner of 0°. Endocarditis vegetation of mitral valve is marked with arrow. (b) Transesophageal echocardiography with a corner of 90°. Endocarditis vegetation of mitral valve is marked with arrow.

**Figure 2:**
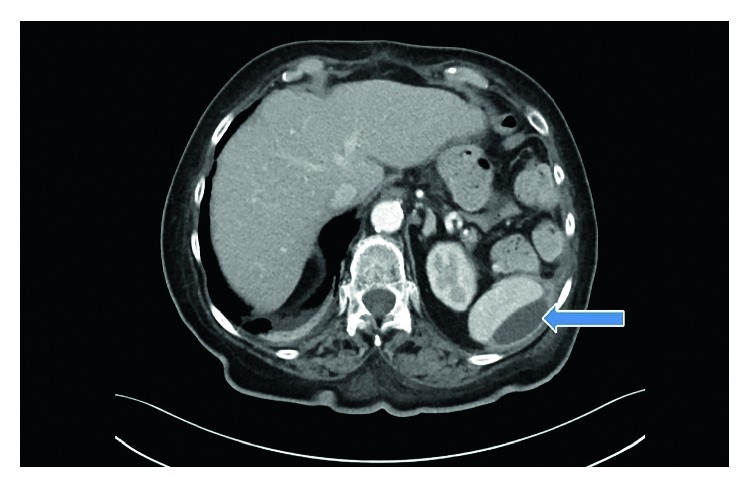
CT scan showing subcapsular fluid formation of the spleen as manifestation of septic herds in endocarditis.

**Figure 3:**
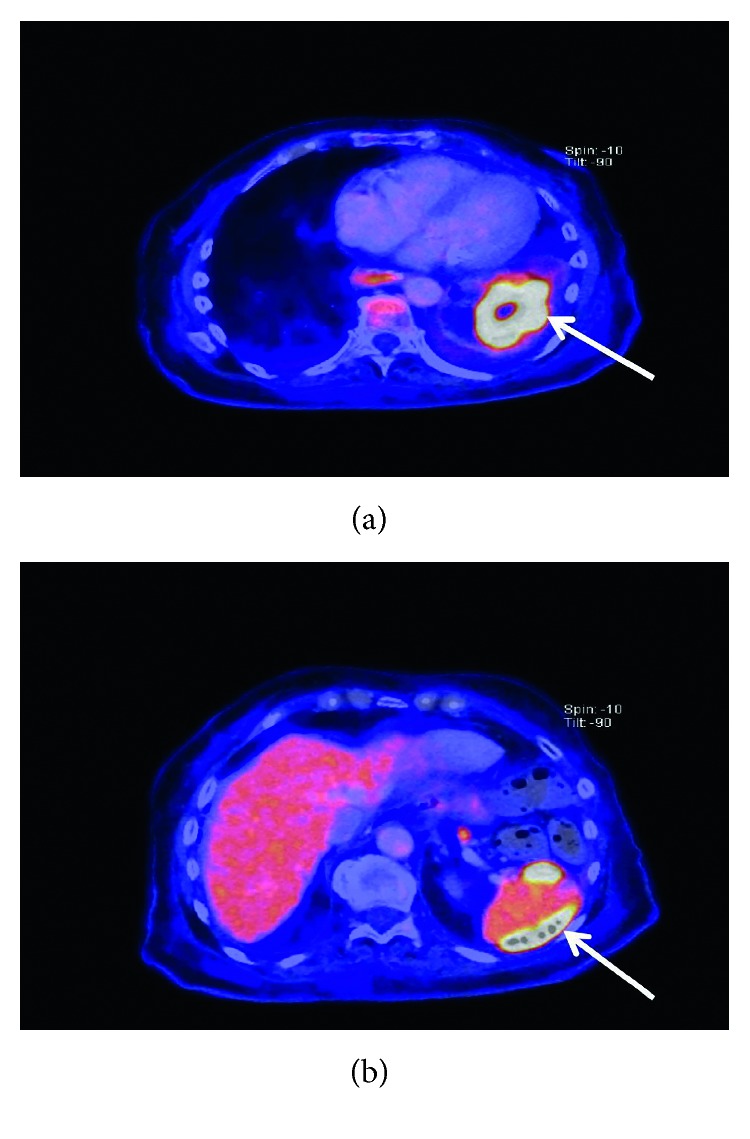
(a) PET/CT scan showing slightly increased 18F FDG uptake of the spleen performed during the antibiotic therapy indicates inflammation. (b) PET/CT scan showing splenic inflammation as well as splenic abscesses.

An 81-year-old Caucasian woman was referred to our department for several weeks of symptoms such as chills, fever, malaise, fatigue, and recurrent tumble despite antibiotic therapy. She was treated prior to admission for approximately two weeks with antibiotics in another hospital. The patient had an important abdominal surgery due to gastric carcinoma three months prior to admission. Gastrectomy, lymphadenectomy, and esophageal stent because of esophageal anastomosis insufficiency were performed during this surgery. The patient had initially presented with chills and recurrent tumble at the family doctor and was at this time admitted in another hospital. With increasing inflammation parameters despite antibiotic therapy and recurrent fever, the medication was changed from amoxicillin to piperacillin/tazobactam on admission. Three independent sets of peripheral blood cultures were obtained before the start of changed antibiotic. All three sets depicted *Lactobacillus* species. The patient denied ingesting any probiotics. We suspected endocarditis because of bacteremia with *Lactobacillus*, recurrent symptoms with worsening of condition, and persistent infection despite antibiotic. On admission, she was febrile to 38.3°C, somnolent, complaining of generalized fatigue and malaise. Her heart rate was 85 bpm, and her blood pressure was 110/75 mmHg. Clinical examination revealed a grade 2/6 systolic murmur loudest at the apex such as no painful haemorrhagic spots (Janeway lesion) on all fingertips of the left hand. Lung auscultation and chest X-ray showed no anomaly. Laboratory studies showed a normocytic anaemia (haemoglobin: 9.0 g/dl; MCV: 85.0 fl), a regular white blood cell count (8.960/*µ*l), reduced platelet count (123.000/*µ*l), elevated C-reactive protein (12.6 mg/dl), and an elevated lactate dehydrogenase (310 U/l). We performed a transesophageal echocardiogram for further diagnosis. It showed degenerative changes of the mitral valve with moderate regurgitation as well as small endocarditis vegetation (Figures 1 and 1 Pronounced vegetation of the mitral valve could not be seen. The susceptibility testing showed that the isolate was resistant to vancomycin. After application of the Duke criteria (one major criterion and three minor criteria were found) [9] in combination with the clinical condition, the diagnosis of endocarditis was more likely. The major criterion was the vegetation and degenerative changes of the mitral valve, and the minor criteria were the three positive blood cultures with *Lactobacillus* species, fever over 38.0°C, and the Janeway lesion found on the left hand. We started an intravenous antibiotic therapy with ampicillin (2 g with six administrations per day) combined with gentamicin (with maximum 80 mg per day) for six weeks. The CT scan on admission revealed subcapsular fluid formation of the spleen (Figure 2). During antibiotic therapy, we performed a PET/CT scan to localize potential inflammatory foci due to embolic complication. Florid inflammatory processes were found in several locations within the spleen (Figures 3(a) and 3(b)). This result was interpreted as peripheral embolization due to endocarditis. We discussed the result of the PET/CT scan with our abdominal/visceral surgeons. They decided upon an operational treatment of the splenic abscesses. The patient underwent an abdominal surgery with splenectomy. Other blood cultures were drawn under the antibiotic therapy. They were all negative without exception. The patient required no mitral valve replacement for cure.

## 3. Discussion

*Lactobacillus* species are an infrequent human pathogen but can induce several infections [5] such as bacteremia, infectious endocarditis, dental caries, and intra-abdominal infections with liver or splenic abscesses [6]. *Lactobacillus bacteremia* with mitral valve endocarditis and splenic abscesses were found in our patient. A gastric surgery took place a few months (three months) prior to endocarditis. It has already been shown in the literature that manipulation of gastrointestinal mucosa induced a temporary bacteremia with *Lactobacillus* [10]. After performing PET/CT scan to localize potential inflammatory foci, we found multiple splenic abscesses. We interpreted this peripheral inflammation as embolization as part of the endocarditis. Prior to the performance of the PET/CT scan, we treated the endocarditis for three weeks with an antibiotic combination of ampicillin and gentamicin. This combination has been established in the past. The endocarditis itself could not be seen in PET/CT. This is explained by the fact that PET/CT was first performed several days after the start of antibiotic therapy, so no inflammatory activity of the mitral valve was detectable.
